# A Novel Microfluidic Device for Blood Plasma Filtration

**DOI:** 10.3390/mi12030336

**Published:** 2021-03-22

**Authors:** Zaidon T. Al-aqbi, Salim Albukhaty, Ameerah M. Zarzoor, Ghassan M. Sulaiman, Khalil A. A. Khalil, Tareg Belali, Mohamed T. A. Soliman

**Affiliations:** 1 College of Agriculture, University of Misan, Al-Amara, Misan 62001, Iraq; 2 Department of Chemistry, College of Science, University of Misan, Maysan 62001, Iraq; 3 Middle Technical University, Technical Institute, Kut 52001, Iraq; ameerah.zarzoor@mtu.edu.iq; 4 Department of Applied Sciences, University of Technology, Baghdad 10066, Iraq; 100135@uotechnology.edu.iq; 5 Department of Medical Laboratory Sciences, Faculty of Applied Medical Sciences, University of Bisha, 255, Al Nakhil, Bisha 67714, Saudi Arabia; kaahmad@ub.edu.sa (K.A.A.K.); Blaly@ub.edu.sa (T.B.); mohamedtalaat25@yahoo.com (M.T.A.S.); 6 Department of Medical Laboratory Sciences, Faculty of Medicine and Health Sciences, University of Hodeidah, Hodeidah 3114, Yemen

**Keywords:** microfluidics, blood plasma filtration, chip extract, blood molecules

## Abstract

The use of whole blood and some biological specimens, such as urine, saliva, and seminal fluid are limited in clinical laboratory analysis due to the interference of proteins with other small molecules in the matrix and blood cells with optical detection methods. Previously, we developed a microfluidic device featuring an electrokinetic size and mobility trap (SMT) for on-chip extract, concentrate, and separate small molecules from a biological sample like whole blood. The device was used to on-chip filtrate the whole blood from the blood cells and plasma proteins and then on-chip extract and separate the aminoglycoside antibiotic drugs within 3 min. Herein, a novel microfluidic device featuring a nano-junction similar to those reported in the previous work formed by dielectric breakdown was developed for on-chip filtration and out-chip collection of blood plasma with a high extraction yield of 62% within less than 5 min. The filtered plasma was analyzed using our previous device to show the ability of this new device to remove blood cells and plasma proteins. The filtration device shows a high yield of plasma allowing it to detect a low concentration of analytes from the whole blood.

## 1. Introduction

Human blood plasma is one of the most convenient and the most important circulating biomarkers sources. Since this free-blood cells matrix has numerous clinically relevant analytes like metabolites, nucleic acids, and proteins, it has become a standard sample for the exclusion or diagnosis of several diseases [1,2]. Moreover, blood plasma is utilized in drug development trials, e.g., for drug monitoring and their metabolites, since it has the drug fraction most relevant to study the pharmacodynamic and pharmacokinetic influences of the drug [3,4]. Plasma transcriptome, proteome, and metabolome studies have increased the spectrum of the diagnostic target for different types of diseases from sepsis to cancer to Alzheimer’s [5,6,7]. Further, foreign nucleic acids as well as antigens and antibodies present in plasma, allow the diagnosis of serious infectious diseases. Additionally, when carrying out plasma analysis in laboratories, blood plasma is useful in the analysis of glucose, total cholesterol, electrolyte concentration, lactate, etc. In clinical chemistry, blood plasma isolation is a necessary step performed, and for the development of miniaturized clinical diagnostic devices, beneficial sample preparation techniques are required [8]. Filtration of plasma from whole blood is very desirable in most cases. Venous blood samples centrifugation is the usual technique to prepare blood plasma with volumes that can be analyzed using highly sensitive methods like LC-MS/MS (liquid chromatography−tandem mass spectrometry). The expansion of blood plasma separation (BPS) based on microfluidics, dealing only with small sample volumes, has rapidly grown in the field of clinical laboratory medicine [9]. This not only increases patient compliance, convenience, and comfort by reducing the amount of blood required to be pulled allowing point of care (POC) sample collection but also permits the analysis to be performed properly at a part of the duration and cost. Moreover, the potential of on-time repetitive sampling, automated parallelization, and portability are other benefits of performing different procedures at the microscale. Microsampling is a procedure for capturing small volumes of biological samples like whole blood from the human body to be analyzed in a minimally invasive method [10]. To deliver sample volumes sufficient to faithfully detect low concentration analytes from limited sample volumes is still the main challenge with microsampling, so a plasma sampling method must be in a high yield to arrive at reasonable levels in the detection of target analytes at low-concentrations and must give pure plasma without blood cells hemolysis or leakage to be clinically pertinent. Furthermore, extraction time is important to reduce the effect of coagulation, particularly when working with fresh samples, e.g., standard finger pricks. To achieve all these requirements, microfluidics is a favorable technology for manufacturing microminiaturized devices that studies fluids’ behavior through micro-channels. There are two types of plasma separation techniques using microfluidics, passive and active. Passive separation techniques demand no external tools, making the devices smaller in size, easier to use, cheaper, and therefore suitable for POC uses [11] while active separation requires exertion of an exterior energy source, e.g., inertial, electric, or acoustic forces [12]. Passive separation techniques can be stored under pressure or driven by capillary forces [13,14,15]. Using capillary forces in plasma separation is more desirable, since it demands neither vacuum packaging, nor degassing of a suction material [16]. The mechanism of separation is generally based on sedimentation, size exception, or a combination of them [17]. Performing plasma separation using sedimentation gives a pure plasma at the time expense. Plasma separation during size-exclusion avoids the time limitation and permits a quick separation. It could be based on porous media like a filter membrane linked to a capillary channel or a membrane stack [18]. Other microfluidic devices for plasma separation have been demonstrated based on diffusion filter/microfilter [19], bends in micro-channels [20], acoustic waves [21], crossflow filtration [22], and dielectrophoresis [23,24]. Major research projects in recent decades have focused on microfluidics, drug, and gene nano delivery systems, tissue engineering, and biosensors due to cost-effectiveness and high performance [25,26,27,28,29]. Recently, we developed a microfluidic device featuring two nano-junctions with different sizes to form a size and mobility trap (SMT) for on-chip filtrate the whole blood from blood cells and plasma proteins, then on-chip extract, concentrate, and separate small molecules from whole blood [30]. The capability of the device was demonstrated for on-site therapeutic drug monitoring (TDM) of aminoglycoside antibiotic drugs within 3 min. However, this device was fabricated for on-chip filtration and analysis of small molecules from whole blood where the ability to collect the filtered blood for use in other applications is not possible through this device. Here, a novel microfluidic device with a nano-junction created by dielectric breakdown is developed for on-chip filtration and out-chip collection of whole blood to be used in other applications as a pure plasma.

## 2. Materials and Methods

### 2.1. Chemicals and Sample Preparation

Fluorescein from Sigma-Aldrich (Sydney, Australia) was prepared in Milli-Q water to get 200 μg/mL solution. Fluorescamine from Sigma-Aldrich was prepared in acetone to obtain 3 mg/mL as a stock solution. Bovine Serum Albumin (BSA) from Sigma-Aldrich was prepared in Milli-Q water 2 mg/mL stock solution and then labeled with Fluorescamine in a ratio 3:1 in borate buffer at pH = 9. Polydimethylsiloxane (PDMS) curing agent and elastomer were purchased from Dow Corning (Michigan, MI, USA). Sodium phosphate monobasic, disodium hydrogen phosphate, and sodium tetraborate were purchased from Sigma-Aldrich (Sydney, Australia) and used for buffer preparation. All solutions were prepared by using Milli-Q water (18 MΩ, Millipore, North Ryde, Australia) purification system. The fresh finger-prick blood used in the experiments was obtained from healthy volunteers approved by the Tasmanian Health and Medical Human Research Ethics Committee, Office of Research Services, the University of Tasmania (Ethics Approval Ref is H0016575).

### 2.2. Device Fabrication

**Figure 1:**
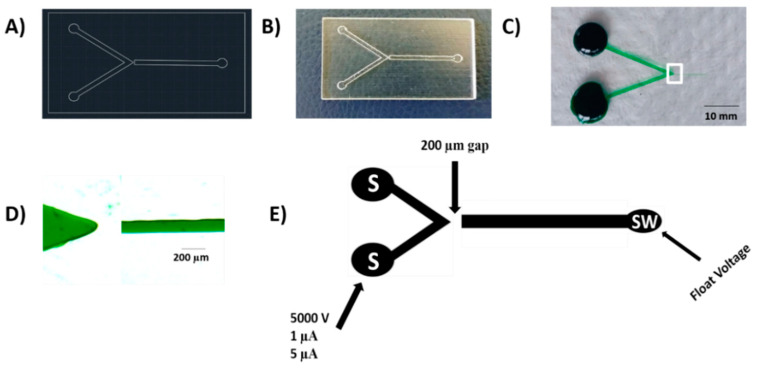
(**A**) An AutoCAD design of the filtration device (**B**) photograph image of the negative 3D printed portable device using an Eden 3D printer (**C**) Photograph image of the hot embossed filtration poly (methyl methacrylate) (PMMA)/adhesive tape device filled with green food dye. Scale bar = 10 mm. (**D**) Zoomed-in image of the V channel and the filtration channel (white box in panel C) filled with green food dye. Scale Bar = 200 µm. (**E**) Schematic of the microfluidic device (dimension not to scale) indicating terminating current and voltages used for generation of the nano-junction Sample (S), and Sample Waste (SW).

Figure 1A presents an AutoCAD design of the device which was then 3D printed using an Eden 3D printer (Figure 1B) to produce a negative template of the device using the previously described process [31]. Then, PDMS was utilized to give the positive master embossing stamp by mixing 210 g in a mass ratio of 5:1 polymer to the elastomer. The PDMS mixture degassed for 15 min and left out for 30 min at room temperature and then poured onto the negative 3D printed template, and then allowed for curing in an oven at 70 °C for at least 12 h. The PDMS positive stamp was cut off and then taken away from the 3D printed template and allowed for thermal age in an oven for 30 min at 250 °C. Then, it used to hot emboss the poly (methyl methacrylate) (PMMA) channel plates (1.5 mm × 50 mm × 75 mm). The final PMMA chips were produced using a hot embossing procedure as reported in our previous work [30]. Briefly, PDMS positive stamp and the blank PMMA plate were placed between two 50 mm × 50 mm × 6 mm glass plates and place into the hot embosser (MTP-8, Tetrahedron, San Diego, CA, USA). Three steps were used in the embossing process. Step 1 was done by increasing the temperature from a rate of (92 °C/min) until the temperature reached (130 °C) and involved maintaining the pressure at 100 lbs. the next step was performed by enhancing the pressure up to 380 lbs at a rate of 75 lbs/min and maintaining the temperature at (130 °C). When the pressure goes up to 380 lbs, those conditions were held for 20 min. In the final step, the temperature was reduced at a rate of 15 °C /min until the temperature reached (60 °C) and involved maintaining the pressure at 380 lbs. The hot-embossed PMMA microchip device was subsequently bonded with single-sided adhesive tape (Tesa SE, Charlotte, NC, USA). An office laminator (Peach 3500, Peach, Switzerland) was then used and the PMMA channel plate and the adhesive tape were sandwiched between two 1 mm stainless steel plates at 20 °C temperature and 5 speed at 4 orientation, with 90-degree clockwise rotation at each pass.

### 2.3. Creation of a Nano-Junction 

The complete microchip device is a hybrid hot-embossed PMMA/adhesive tape with a photo of the device shown in Figure 1C and a zoomed-in image of the V-channel and the filtration channel in Figure 1D. The V-channel was 500 μm wide and the filtration channel was 50 μm. The V-channel tip was separated from the filtration channel by a 200 μm gap of PMMA to form the filtration nano-junction. This gap was chosen to allow for lengthy use of higher voltages through the filtration without the secondary breakdown’s danger. To form the nanochannel, the V-channel and filtration channel were filled with the breakdown electrolyte, be composed of 10 mM phosphate buffer, pH 11. The filtration nano-junction was created by applying a high voltage of 5000 V to the V-channel whilst the filtration channel was kept grounded Figure 1E. Previously, the high voltage breakdown was 4000 V instead of 5000 V in this work, lower than what was performed in this work. This different voltage in the filtration PMMA devices could be regarding the difference in the gap distance between the separation channel in the double V device and the filtration channel here. The current limit was varied from 1 μA to 5 μA by utilizing an in-house adjustable power supply, controlling by LabView HV V.6 program (National Instrument, Austin, TX, USA) to realize the best repeatability. The V-channel and the filtration channel were cleaned and refilled with the experimental solutions.

### 2.4. Device Operation and Experimental Practice

**Figure 2:**
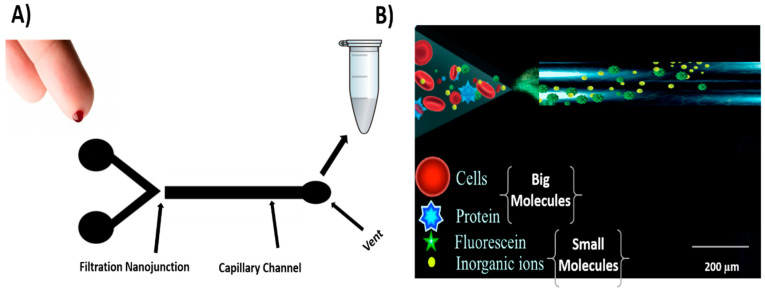
(**A**) Schematic of the operation of the microfluidic device (dimension not to scale) indicating the filtration process and (**B**) illustration of the filtration device concept. Nanochannel was formed by controlled dielectric breakdown of the tip of the V-channel and the filtration channel. Large molecules are blocked (Cells and Proteins) while small molecules pass the filtration channel.

Figure 2A,B show the operation of the filtration device, where the blood volume from a fresh finger prick was controlled by pipetting 50 μL of whole blood to the devices to ensure result comparability and evaluate the filtration method. Pipetting was carried out using an autopipette, and the experiments were performed at room temperature to reduce blood evaporation through the filtration process. A simple technique was used in this approach by pushing the whole blood using the autopipette from the V-channel to the filtration channel through the nanochannel and then a hand syringe-vacuum was used to collect the filtered plasma blood in a high yield. All experiments were performed with a Nikon Eclipse Ti−U inverted fluorescence microscope (Nikon Instruments Inc.) worked with NIS-Elements BR 3.10 software (Melville). A filter cube (Semrock, Rochester) composed of an excitation band-pass filter (488 ± 10 nm), emission filter (520 ± 10 nm), and dichroic mirror to deflect the broadband light source to a 20× objective was used to perform all the experiments. Fluorescence images were carried out using a high-definition color charge-coupled device camera (Digital Sight DS. Filc, Nikon, Japan). A photon multiplier tube (PMT) (Hamamatsu Photonics KK, Hamamatsu, Japan) linked to the microscope was used to record the electropherograms. The PMT was linked to an Agilent 35900E A/D box to allow data collection with the Chemstation software (Agilent Technologies, Waldbronn, Germany). An electrical potential was applied by using an in-house 4-channel (0–5 kV) dc power supply to each reservoir using a custom-designed interface connected to 2 platinum electrodes.

## 3. Results and Discussion

### 3.1. Filtration and Permeability Studies

**Figure 3:**
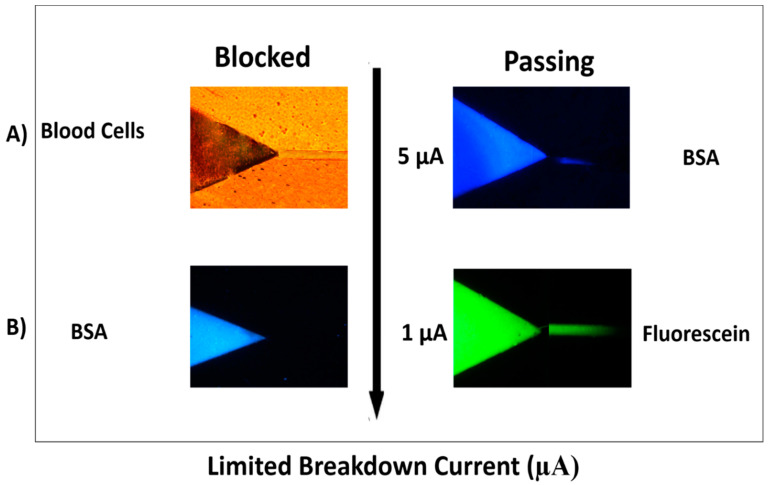
Screenshots presenting the restriction (left column) and passing (right column) of different molecules through nano-junctions formed under different conditions. (**A**) shows the use of 5 μA to create nanochannels which transported Bovine Serum Albumin (BSA) (blue, right) and restricted Blood Cells (left); (**B**) shows the use of 5 μA to create nanochannels which restricted BSA (blue, left) and transported fluorescein (green, right). The nanochannels formed using a breakdown electrolyte of 10 mM phosphate buffer, pH = 11, and terminating currents of 5 and 1 μA. Images on the left show blocked transport, while those on the right show the permeability of different molecules. Scale Bar = 200 μm.

Previously, we examined the relevance between the current limit and nano-junction permeability in the double V hot-embossed PMMA device through different breakdown experiments using different charge and size analytes. Briefly, using a current limit of 5 μA, the resulted nanochannels restricted blood cells (6–8 μm) [32], and R-phycoerythrin (RPE) (<10 nm in size) [33] from passing the separation channel while bovine serum albumin (BSA) (2–4 nm) [34] labeled-fluorescamine allowed to pass the separation channel. Reducing the current limit to 1 μA, blocked the BSA, but allowed the transport of anionic small molecules, such as fluorescein (1 nm) [34] and drugs. The SMT in our previous work is based on ions’ favorable electrokinetic transport through the resulting nanochannels. The extraction nanochannel was created by applying for a 1 μA permit the transport small ions (<1000 Da) during the nano-junction, whilst blocking the transport of plasma proteins and blood cells. The concentration nanochannel between the V-sample channel and the separation channel was created by applying a 0.1 μA current limit to avoid target analytes transport but allows the transfer of small inorganic ions to make a sample desalting. Both the extraction and concentration nanochannels make the SMT in target analytes which can purify, concentrate, desalt, and separate the sample. Here, we build on our previous breakdown work and implement our new design. The results were similar to those reported in our previous work and summarized in Figure 3. We have formed two types of nano-junctions for the filtration process. First, since the current limit was set at 5 μA (Figure 3A), the produced nanochannels blocked blood cells from infiltrating the filtration channel, while allowing the transport of proteins like BSA into the filtration channel. For protein recovery assessment, the termination current was reduced to 1 μA (Figure 3B) which blocked the BSA but permitted the electrophoretic transport of small molecules (<1000 Da) such as fluorescein and drugs. Based on the transport results of different sized molecules (fluorescein 1 nm, BSA 2–4 nm, and Blood cell 4–6 µm), the size of fabricated nanochannels can be estimated with this method.

### 3.2. Analysis of Filtered Blood Plasma

**Figure 4:**
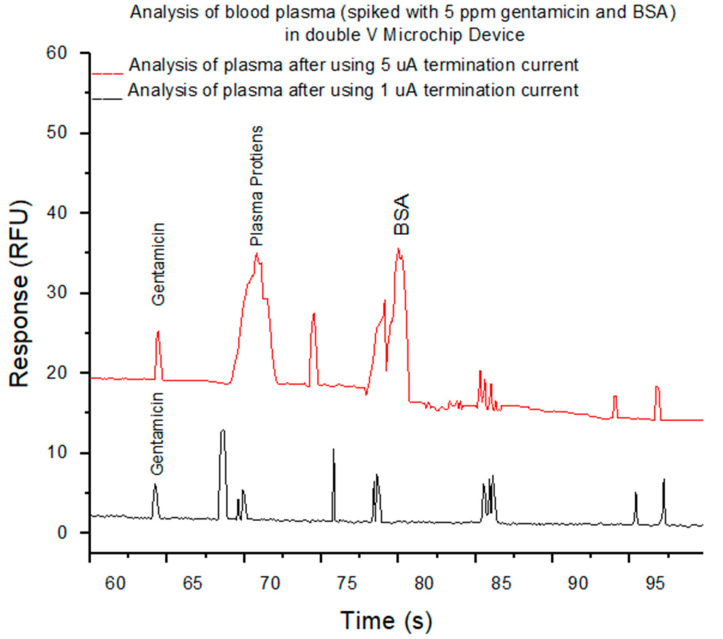
Electropherograms show the analysis of filtered blood spiked with 5 ppm gentamicin and BSA after labeling with fluorescamine in size and mobility trap (SMT) device after using a current limit of 5 uA (red trace) and 1uA current limit (black trace). The background electrolyte (BGE) in the separation channel, was 100 mM phosphate buffer, pH 11.5, with 0.5% HPMC, while V-sample waste channel was 10 mM phosphate buffer, pH 11.5. Applied voltages used in SMT device for extraction and concentration were −200, −850, −600, and +650 V for 60s, and separation process were −250, +250, +2200, and −500 V at reservoirs B, S, BW, and SW, respectively.

**Figure 5:**
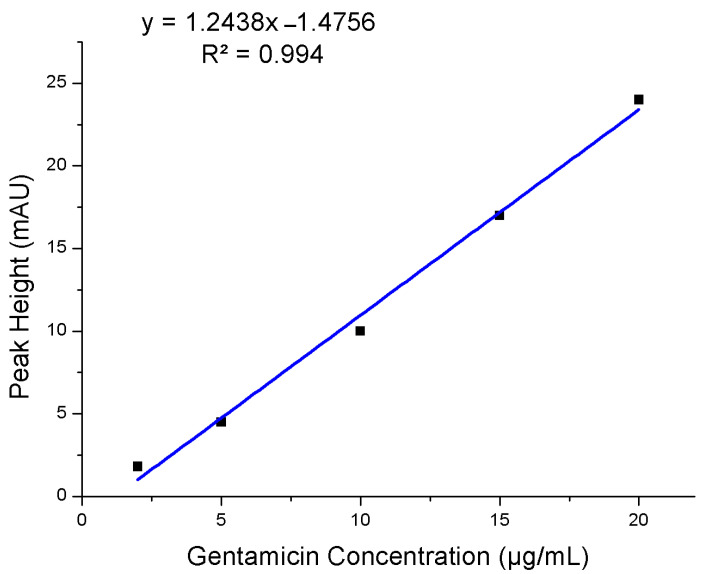
The linear calibration curve for Gentamicin from whole blood.

The color of the plasma was properly similar to centrifuged plasma treated with EDTA, seemed clear without a pink trace in all filtered blood plasma samples which mentioned that the separation of plasma caused no or a little lysis of erythrocytes as clearly seen in Figure 3A. The purpose of the PMMA hot-embossed filtration device is to purify the whole blood from cells and plasma proteins and allow to pass small molecules like pharmaceuticals with filtered plasma. To evaluate the protein recovery assessment, centrifuged plasma relative to filtered plasma spiked with 5 ppm gentamicin and BSA was assessed for both venous blood samples treated with EDTA and filtered finger-prick blood. Two types of filtered plasma using our filtration device were used after applying 5 µA and 1 µA termination currents to create different sized nano-junctions comparing to an EDTA-treated centrifuged plasma. These all were analyzed using our previous SMT device after labeling the plasma with fluorescamine reagent and followed by their separation using electrophoresis. Electrophoresis is a separation approach in which particles, molecules, and ions are separated in a conducting liquid medium using an electric field. All primary amines found in the plasma including those on proteins, amino acids, urea, etc., will be reacted with fluorescamine to produce a fluorescent complex within seconds. Figure 4 shows the electrophoretic separation of filtered blood plasma labeled with fluorescamine after using two different nano-junctions. Filtering whole blood using a device with a termination current of 5 µA and analyzed with the double V-device gives a pure peak for gentamicin, but plasma proteins and BSA are observed. This indicates plasma proteins can pass through the nano-junctions, while blood cells cannot. In contrast, using a termination current of 1 µA gives pure separations due to the observing of small molecules without interfacing and also the removal of some larger proteins, allowing full quantitation of some small molecules in the blood, with a relative standard deviation (RSD%) = 7.5% for gentamicin (n = 3 devices) and with a high recovery efficiency for Gentamicin 94%, and a linear calibration curve of gentamycin from blood is shown in Figure 5. In comparison to other microfluidic devices, the presented device display that the microchip enables the low-cost, rapid, and operationally straightforward separation of plasma from whole blood; also, it demands a small amount of blood to give results within less than 5 min. Therefore, the device provides a favorable solution for biochemical assays.

## 4. Conclusions

In this work, we have introduced a new device for blood plasma separation using a piece of PMMA microfluidic device. The PMMA filtration device dominant dielectric breakdown enabling the pore size tuning of nano-junctions and thus their permeability for different sized molecules. The creation of nanochannels using dielectric breakdown is fast, simple, and does not depend on valves or pumps. This manner enabled the analysis of the filtered blood which was labeled with fluorescamine. The device is portable and can be offered for the implementation of blood plasma instead of centrifugation process and in POC devices as the device could be engineered into a hand-held portable device and integrated with a hand-held reader for on-site testing and can tackle issues regarding sample storage, stability, and shipping and could be a fully separate plasma sampling device for POC.
